# Supplementary material for: Effects of mobility restrictions during COVID19 in Italy
Source: Sci Rep. 2021 Nov 8;11:21783. doi: 10.1038/s41598-021-01076-x (PMC8575918; doi:10.1038/s41598-021-01076-x)
Supplement: Supplementary file 1 — Supplementary Information. [file 41598_2021_1076_MOESM1_ESM.pdf]

Effects of mobility restrictions during COVID19 in Italy - Supplementary

Alex Smolyak[1,*], Giovanni Bonaccorsi[2], Andrea Flori[2], Fabio Pammolli[2,3] Shlomo Havlin[1]

[1] Department of Physics, Bar-Ilan University, Ramat-Gan 52900, Israel;
[2] Impact, Department of Management, Economics and Industrial Engineering, Politecnico di Milano
[3] SIT, Schaffhausen Institute of Technology, Schaffhausen
[*] Correspondence to alex.smolyak@gmail.com

1. Additional Network statistics

Our data consists of tiles, as defined by Facebook which we identify as nodes, and the people traveling between those tiles form the edges. There are app. 5000 nodes or tiles throughout our data set, covering about 80% of Italy's nominal territory. Of those, approximately 3700 take part in non-local interactions (i.e. where there is mobility between the tiles and not only within). Fig. S1 details the joint distributions of the edge weights and distances aggregated for the periods defined in the text.

2. Numerical fits

While the distribution of the distances shows an approximate fit to a power law distribution over several orders of magnitude, the case for the weighted degree distribution is not as evident. Another possible candidate is the Stretched Exponential (Weibull) distribution, with the CDF parameterised as $F = 1 - e^{-(\lambda x)^{\beta}}$. Previous research suggested mechanisms for the existence of such distributions in empirical data (c.f. Refs. [1, 2], and empirical experiments in Ref. [3])

3. Degree Distributions

In the main text we focus attention on edges and their distributions, but the discussion on GDP forecasting deals with average weighted degrees. Here we wish to elaborate of the degree distribution in mode detail. Fig. S3 shows the weighted degree distribution outright (complimenting the CCDF view in the main text). It is clear the distributions have much heavier tails than the ER case. As such, the average degree tends to be strongly affected by the largest values, and does not represent well the typical degrees. However, it is quite likely that the highest weights or degrees contribute most to the economy and flow of activity, therefore the relation between GDP and the average degree may very well depend on the higher end of the degree distribution more than on the typical degree.

4. Impact distribution

The panels in Fig. 5 in the main text show the quality of the linear fit for the impact incurred by all provinces. In order to expand on the inhomogeneity that *is* present, we bring here (Fig. S4) the distributions of each of the periods' impact. A typical impact is seen in all three relatively narrow distributions, however, values around the peak show the provinces deviating from the linear relation.

5. Failing edges as criticality

As was shown in Fig. 4 in the main text, the critical levels q_c for for the fragmentation of the GCC during lockdown are around the value of 0.2. Here we show the failing edges and their respective weights during each of the reported periods. As Fig. S5 shows, both the number of nodes for which some of the edges fail, and the magnitude of failure (i.e. how many edges each node loses) highlight the loss of edges leading to the fragmentation. Top left panel (pre-lockdown) reveals a very strong network where the relatively low levels of q_c inflict almost no damage. During lockdown, as can be seen in the top right figure, and summarized in panel (a) of Fig. 6, multiple edges fail, many of them carrying substantial weight. Lastly, we note again the qualitative difference between the post-lockdown state as compared to the pre-lockdown, where we see many more edges failing at the same, previously almost imperceptible, level.

6. Correlation

As is often the case, care must be taken when calculating correlation. It shows linear dependence between two variables, and as such caries interesting information but also potential for error. One such error may occur if the

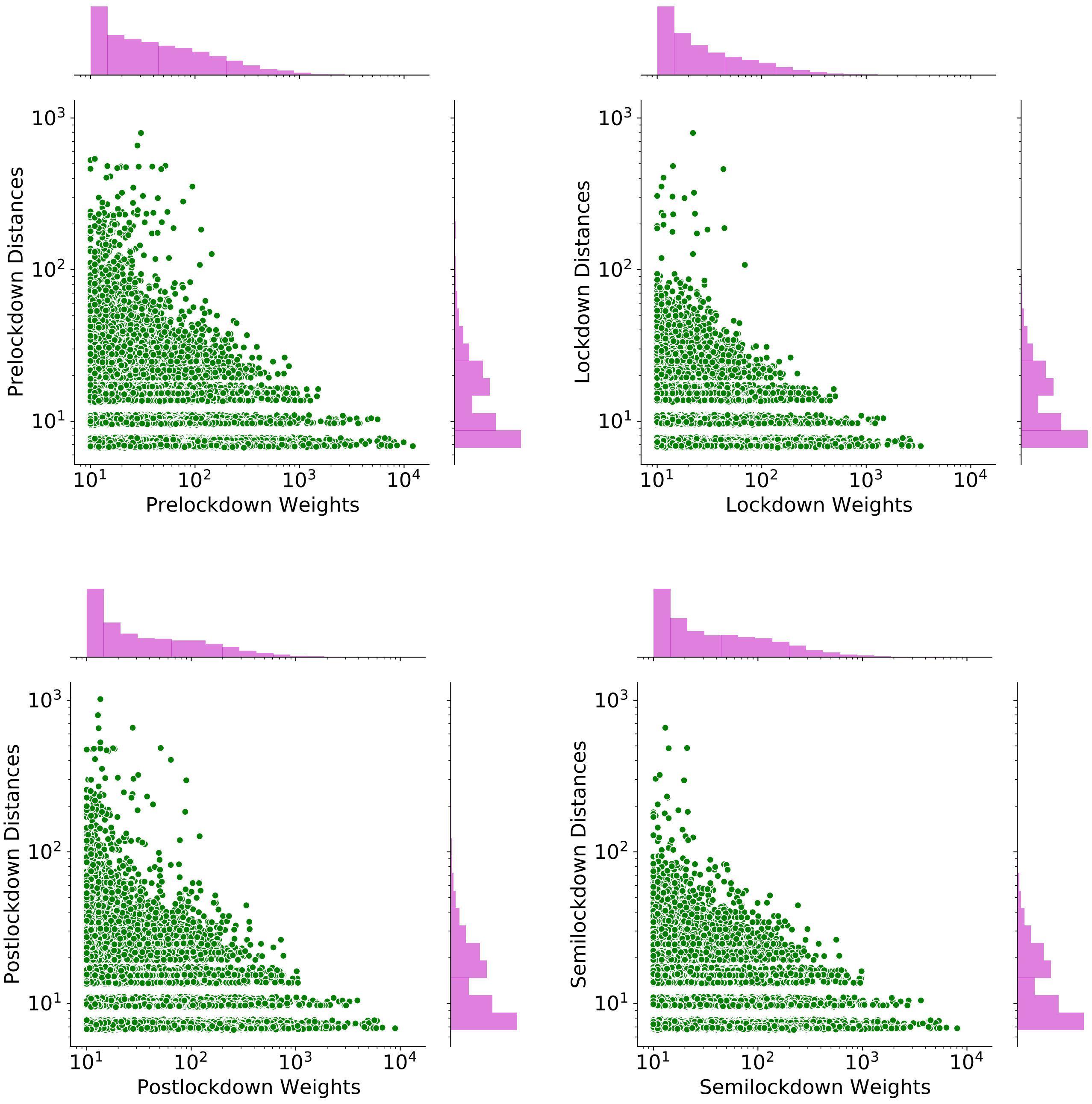

Figure S1: Joint distributions of weights and distances. Clockwise from top left, the distribution before lockdown, under full restrictions, with partial restrictions lifted and after the full lift of limitations.

variables in question contain outliers that are far from most other values. This may lead to artificially shifting the correlation line toward the outlier to minimize its square penalty. Thus, while mathematically correct, the best-fitting linear relation between two variables will not actually represent the relation between the majority of the data. Here we are dealing with economic variables that tend to be heavy-tailed distributed, and thus may suffer from the above-mentioned outliers. When we examine the data, we see both mobility and GDP data are indeed heavy-tailed. In order to avoid outlier pitfalls we apply the log transform to both data sets, and indeed the transformed data is indeed very close to being normally distributed, as Fig. S6 shows. We then are able to calculate the correlation between the transformed data sets without concern of outliers. Generally, because the logarithm is a nonlinear transform, we should not expect for the correlation to stay the same as for the original data. In fact, we would expect the values to change. In our case, however, the correlation between the raw data and its log transformed version is minor (0.66, 95% CI [0.54 0.75] raw, 0.64, 95% CI [0.51 0.74] transformed). This means that the data points at the right edges of our distributions still behave as expected from the center-mass samples.

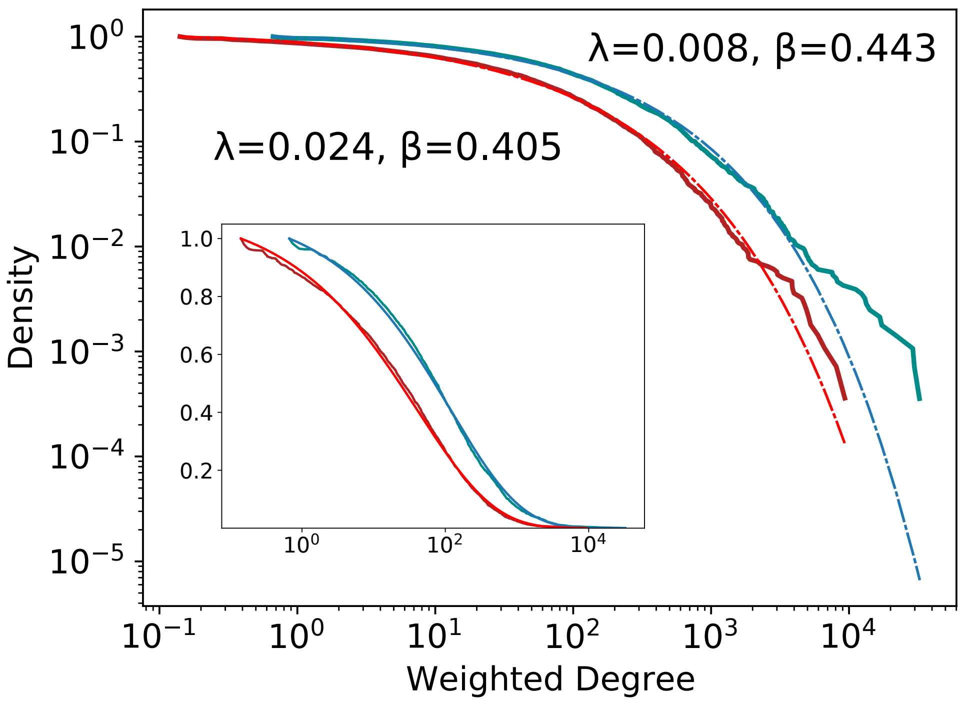

Figure S2: The log-log Complementary CDF plot of the weighted degree distribution with a stretched exponential fit (insert - semilog plot of the same distribution, showing the quality of the fit for the low values as well)

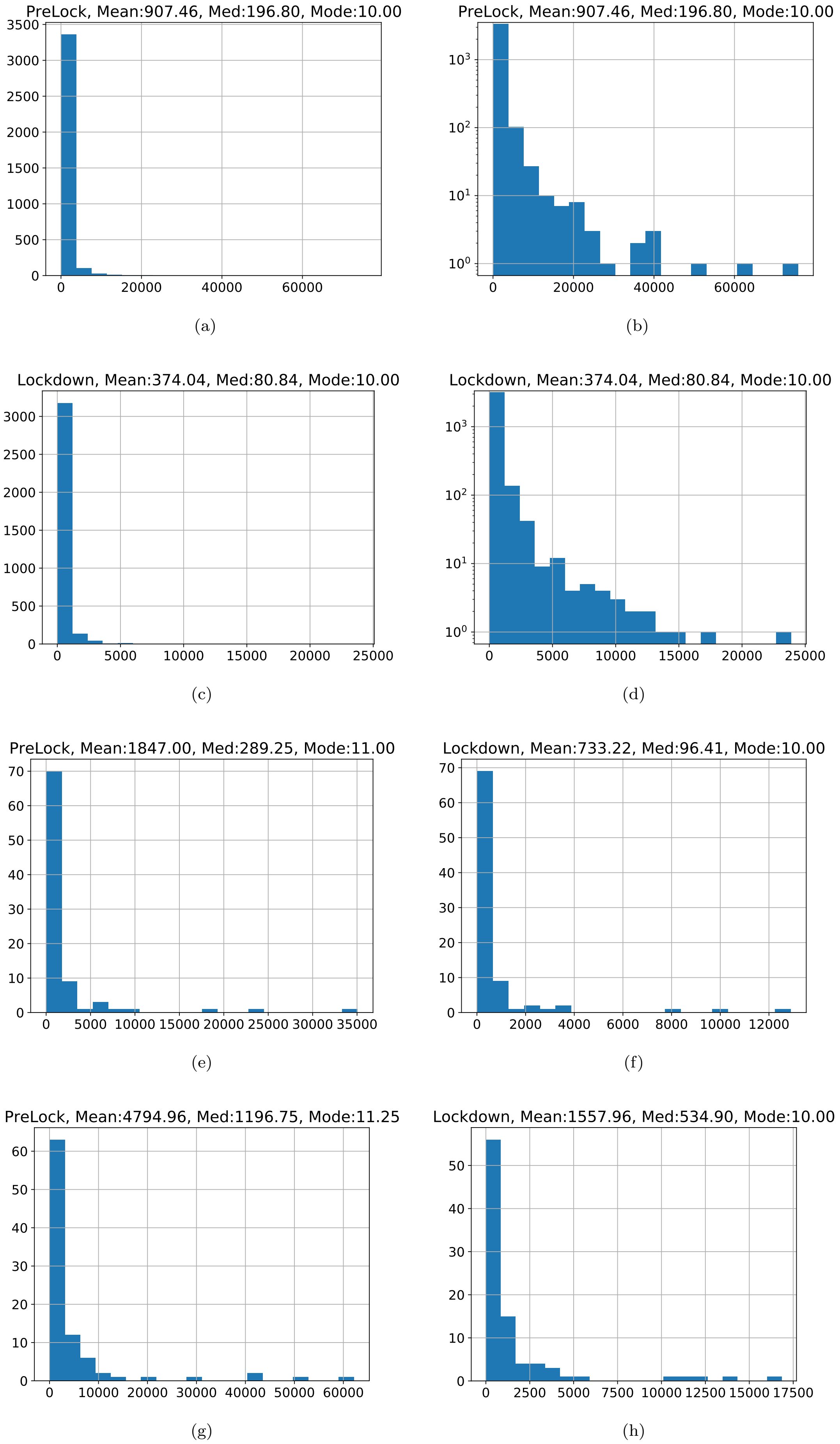

Figure S3: Degree distributions of the networks and some provinces. (a),(b) Histogram and semilog histogram of the network's degree distribution before the lockdown; (c),(d) Same as (a),(b) during lockdown; (e),(f) Degree histogram before and during Lockdown for the province of Turin; (g),(h) Same as (e), (f) for the province of Rome

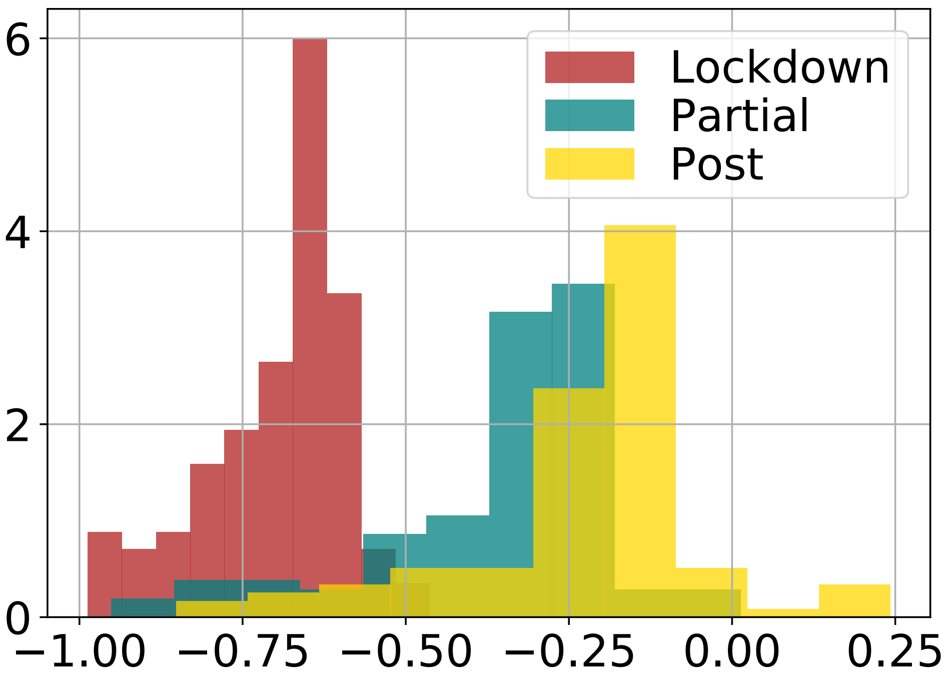

Figure S4: The colors, matching the color scheme in the main text, show the impact is indeed centered around similar values for the lockdown and following stages, but those values are not the whole story. Specifically, for the post-lockdown stages, the distribution is broader, and thus, as the main text explains, is the variation in the economic impact.

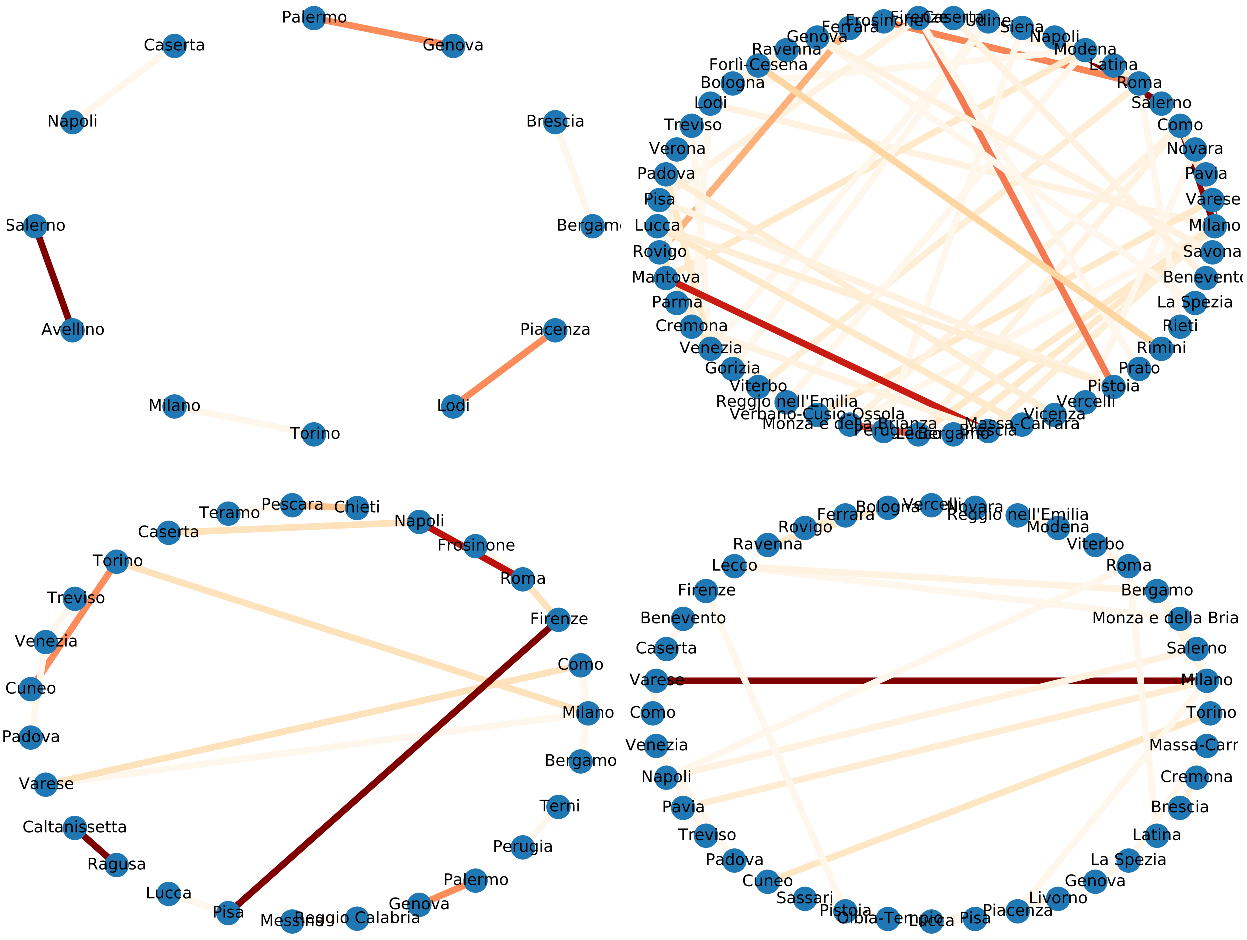

Figure S5: As before, clockwise from top left, the pre-lockdown, lockdown, partial limitations and post lockdown states. The edges in the figure are the ones removed at this level of $q_c = 0.22$. Edge color is relative to the other edges for the same period.

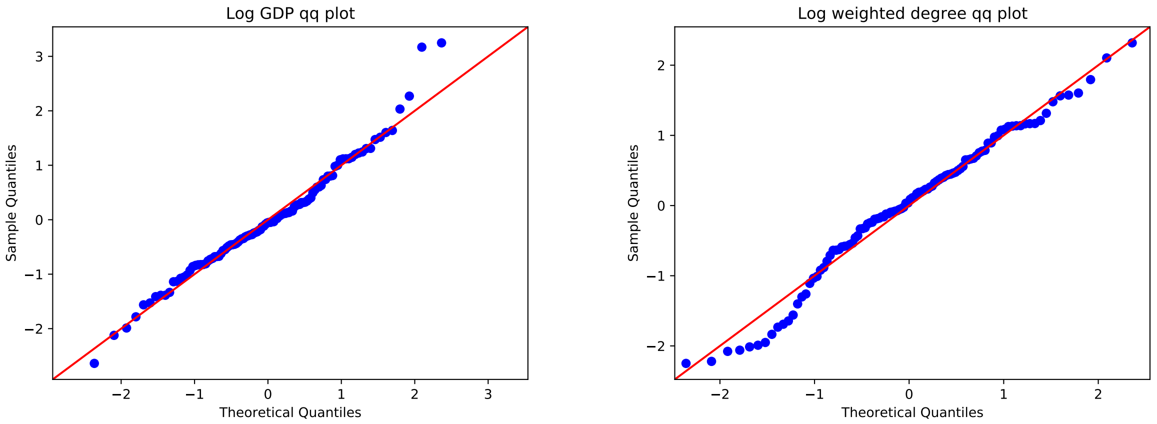

Figure S6: We show here the qq-plots of the log-transformed data. The 45° line shows where an ideal normal distribution would lie, to which our data corresponds well after the log-transform

7. References

1. Laherrere, J. & Sornette, D. Stretched exponential distributions in nature and economy: "fat tails" with characteristic scales. *The European Physical Journal B-Condensed Matter and Complex Systems* **2,** 525–539 (1998).
2. Frisch, U. & Sornette, D. Extreme deviations and applications. *Journal de Physique I* **7,** 1155–1171 (1997).
3. Alstott, J., Bullmore, E. & Plenz, D. powerlaw: a Python package for analysis of heavy-tailed distributions. *PloS one* **9,** e85777 (2014).
